# Neurodevelopmental multimorbidity and educational outcomes of Scottish schoolchildren: A population-based record linkage cohort study

**DOI:** 10.1371/journal.pmed.1003290

**Published:** 2020-10-13

**Authors:** Michael Fleming, Ehsan E. Salim, Daniel F. Mackay, Angela Henderson, Deborah Kinnear, David Clark, Albert King, James S. McLay, Sally-Ann Cooper, Jill P. Pell

**Affiliations:** 1 Institute of Health and Wellbeing, University of Glasgow, Glasgow, United Kingdom; 2 Information Services Division, Edinburgh, United Kingdom; 3 ScotXed, Scottish Government, Edinburgh, United Kingdom; 4 Department of Child Health, University of Aberdeen, Aberdeen, United Kingdom; Stellenbosch University, SOUTH AFRICA

## Abstract

**Background:**

Neurodevelopmental conditions commonly coexist in children, but compared to adults, childhood multimorbidity attracts less attention in research and clinical practice. We previously reported that children treated for attention deficit hyperactivity disorder (ADHD) and depression have more school absences and exclusions, additional support needs, poorer attainment, and increased unemployment. They are also more likely to have coexisting conditions, including autism and intellectual disability. We investigated prevalence of neurodevelopmental multimorbidity (≥2 conditions) among Scottish schoolchildren and their educational outcomes compared to peers.

**Methods and findings:**

We retrospectively linked 6 Scotland-wide databases to analyse 766,244 children (390,290 [50.9%] boys; 375,954 [49.1%] girls) aged 4 to 19 years (mean = 10.9) attending Scottish schools between 2009 and 2013. Children were distributed across all deprivation quintiles (most to least deprived: 22.7%, 20.1%, 19.3%, 19.5%, 18.4%). The majority (96.2%) were white ethnicity. We ascertained autism spectrum disorder (ASD) and intellectual disabilities from records of additional support needs and ADHD and depression through relevant encashed prescriptions. We identified neurodevelopmental multimorbidity (≥2 of these conditions) in 4,789 (0.6%) children, with ASD and intellectual disability the most common combination. On adjusting for sociodemographic (sex, age, ethnicity, deprivation) and maternity (maternal age, maternal smoking, sex-gestation–specific birth weight centile, gestational age, 5-minute Apgar score, mode of delivery, parity) factors, multimorbidity was associated with increased school absenteeism and exclusion, unemployment, and poorer exam attainment. Significant dose relationships were evident between number of conditions (0, 1, ≥2) and the last 3 outcomes. Compared to children with no conditions, children with 1 condition, and children with 2 or more conditions, had more absenteeism (1 condition adjusted incidence rate ratio [IRR] 1.28, 95% CI 1.27–1.30, *p <* 0.001 and 2 or more conditions adjusted IRR 1.23, 95% CI 1.20–1.28, *p <* 0.001), greater exclusion (adjusted IRR 2.37, 95% CI 2.25–2.48, *p <* 0.001 and adjusted IRR 3.04, 95% CI 2.74–3.38, *p <* 0.001), poorer attainment (adjusted odds ratio [OR] 3.92, 95% CI 3.63–4.23, *p <* 0.001 and adjusted OR 12.07, 95% CI 9.15–15.94, *p <* 0.001), and increased unemployment (adjusted OR 1.57, 95% CI 1.49–1.66, *p <* 0.001 and adjusted OR 2.11, 95% CI 1.83–2.45, *p <* 0.001). Associations remained after further adjustment for comorbid physical conditions and additional support needs. Coexisting depression was the strongest driver of absenteeism and coexisting ADHD the strongest driver of exclusion. Absence of formal primary care diagnoses was a limitation since ascertaining depression and ADHD from prescriptions omitted affected children receiving alternative or no treatment and some antidepressants can be prescribed for other indications.

**Conclusions:**

Structuring clinical practice and training around single conditions may disadvantage children with neurodevelopmental multimorbidity, who we observed had significantly poorer educational outcomes compared to children with 1 condition and no conditions.

## Abstract 2

Michael Fleming and colleagues investigate educational outcomes among children with comorbid depression, attention deficit hyperactivity disorder, autism, or intellectual disability.

## Author summary

**Why was this study done?:**

- Children with neurodevelopmental conditions experience difficulties at school.
- Multiple neurodevelopmental conditions commonly coexist together.
- We investigated prevalence of neurodevelopmental multimorbidity in Scottish schoolchildren and their educational outcomes compared to peers.

**What did the researchers do and find?:**

- We linked Scotland-wide health and education data together to identify neurodevelopmental multimorbidity among children attending school in Scotland between 2009 and 2013, inclusive.
- We identified children treated for depression, attention deficit hyperactivity disorder (ADHD), autism, and intellectual disability and defined comorbidity as 2 or more of those conditions existing together.
- Compared to children with no conditions, children with 1 condition, and children with 2 or more of these conditions, experienced increased school absenteeism and exclusion, poorer exam attainment, and increased unemployment.

**What do these findings mean?:**

- Structuring clinical practice and training around single conditions may disadvantage children with multimorbidity, who we observed in our study to have significantly increased risk of poor educational outcomes compared to children with 1 condition and no conditions.

## Introduction

Multimorbidity in adulthood has attracted increasing recognition and was highlighted by the Academy of Medical Sciences as a global priority for health research [1]. In comparison, childhood multimorbidity has received little attention. Indeed, children with neurodevelopmental disorders such as attention deficit hyperactivity disorder (ADHD) [2–4], autism spectrum disorder (ASD) [5, 6–8], depression [9, 10], and intellectual disability [11–13] appear to be at particular risk of multimorbidity, and in 2010, the phrase Early Symptomatic Syndromes Eliciting Neurodevelopmental Clinical Examination (ESSENCE) was first used in recognition of the frequent coexistence of childhood neurodevelopmental disorders [14]. Pre-school children with ASD, for example, have been reported to have a mean of 3.2 coexisting neurodevelopmental disorders, including ADHD and intellectual disability [5], and comorbidities such as anxiety and obsessive-compulsive disorder (OCD) are also common [6–8]. Common conditions existing alongside ADHD include depression, intellectual disability, anxiety, ASD, conduct disorder, oppositional defiant disorder, tics, and Tourette [2–4, 15–17]. Studies on children with intellectual disabilities have reported a range of comorbid internalizing and externalizing mental health conditions such as ADHD and depression [11–13]. Depression commonly occurs with various anxiety disorders [9, 10]. Furthermore, there is emerging evidence of pleiotropy [18]. This underlying predisposition to multiple conditions is important because clinical training, clinical pathways and interventions tend to be structured around single disorders. For example, in the United Kingdom, in “core” psychiatric training (the first 3 years of psychiatric training) and in “higher” specialist training in child and adolescent psychiatry (a further 3 years’ training to complete specialist training and be eligible for a consultant position), the core intended learning objectives that the Royal College of Psychiatrists require to be met specify learning on all the individual common psychiatric conditions, comorbidity with physical conditions, but not comorbidity of psychiatric conditions [19, 20]. This incongruent approach may fail to fully recognise and address these children’s complex needs and their propensity to disease–disease, drug–disease, and drug–drug interactions.

Chronic neurodevelopmental disorders have been associated with poorer school outcomes [21]; however, previous studies reporting educational outcomes for children with neurodevelopmental disorders have tended to focus only on individual conditions such as ASD [22, 23], ADHD [24, 25], depression [26, 27, 28], and intellectual disability [29, 30]. As a result, there is a paucity of literature reporting outcomes for children with coexisting neurodevelopmental conditions [31–33]. Previous studies investigating singular or comorbid neurodevelopmental conditions have used small cohorts, with only a handful of larger studies focussing on the educational outcomes associated with single conditions [24, 28, 34]. Small studies that have investigated school outcomes of children with co-occurring conditions have mainly focussed on comorbid ADHD with respect to academic attainment, with few investigating school grades and most using battery tests [32] or parental, teacher, or pupil feedback [31, 33, 35].

The aim of this study was to address a gap in the literature by determining the countrywide prevalence of neurodevelopmental multimorbidity among Scottish schoolchildren and investigating a wider range of associated, administratively recorded, educational outcomes: absenteeism, school exclusion, exam grades, and unemployment. We hypothesised that children with neurodevelopmental multimorbidity would have poorer outcomes compared to children with 1 condition and no conditions.

## Methods

This study is reported as per the Strengthening the Reporting of Observational Studies in Epidemiology (STROBE) guideline (S1 STROBE Checklist). While we did not publish an analysis plan, our analyses were planned in advance of the research team accessing any data. We subsequently added formal mediation analyses and sensitivity analyses additionally adjusting for comorbid physical health conditions and additional support needs at the request of the reviewers.

### Databases

Four Scotland-wide education databases were accessed via the Scottish Exchange of Educational Data (ScotXed) Unit in the Learning and Justice Directorate of the Scottish Government: the annual Pupil Census, which includes pupils’ age, sex, ethnicity, and records of additional support needs (referred to as special educational need in the Pupil Census); attendance data including absences and exclusions which are routinely collected prospectively and appended to the census at the end of each school year; the Scottish Qualifications Authority (SQA) database, which records school examination results; and the School Leaver Database, which records the education/employment status of pupils 6 months after leaving school. Two Scotland-wide health databases were accessed via the Information Services Division (ISD) of the NHS National Services Scotland: maternity inpatient and day case records (Scottish Morbidity Record [SMR] 02), which collects maternity and obstetric data pertaining to mother and child, and the Prescribing Information System (PIS), which contains information on all medicines dispensed in the community in Scotland. These 6 databases were linked at an individual level using a mixture of deterministic and probabilistic matching. Health records in Scotland contain a Community Health Index (CHI) number, a unique 10-digit patient identifier enabling health records for the same person to be linked together. The CHI database is a register of all patients in NHS Scotland. Education records from each pupil census were probability matched against the CHI database using sex, date of birth, and postal code, and the matched CHI numbers were then used to deterministically match education to health data. The methodology has been described in greater detail elsewhere [24, 28, 34, 36].

### Case ascertainment and definition

The annual Pupil Census was used to identify any children with a record of additional support needs due to ASD or intellectual disabilities (referred to as learning disability in the Pupil Census). ADHD and depression were ascertained using PIS data. ADHD was defined as receipt of at least 1 encashed prescription for atomoxetine, dexamphetamine, lisdexamphetamine, or methylphenidate in a school year. Depression was defined as receipt of at least 1 encashed prescription for a selective serotonin reuptake inhibitor (SSRI), mirtazapine, venlafaxine, or a tricyclic antidepressant, in a school year. Multimorbidity was defined as 2 or more of any of these conditions being present in the same child.

### Inclusion and exclusion criteria

Our cohort comprised children who were included on any of the pupil censuses undertaken between 2009 and 2013 inclusive and who therefore attended a primary, secondary, or special school in Scotland—maintained by a local authority—over that time period. Only pupils between the ages of 4 and 19 years at the time of the Pupil Census were included. Inclusion was restricted to singleton births due to difficulties linking the correct child for multiple births of the same sex (S1 Fig).

### Outcomes

The study included 4 outcomes: annual number of days absent from school, annual number of school exclusions (also referred to as expulsions or suspensions) for challenging/disruptive behaviour, academic attainment, and subsequent unemployment. The latter 2 outcomes were restricted to pupils who left school during the study period. From 2010 onwards, the Scottish Government only collected absence and exclusion data every 2 years, therefore these data were only available for 2009, 2010, and 2012 and were not collected in 2011 or 2013. Attainment was derived from the total number of examination awards achieved by each pupil at each level of the Scottish Credit Qualifications Framework (SCQF) recorded by the SQA [37]. Attainment was dichotomised as low attainment (≥1 at SCQF level 2, ≥5 at SCQF level 3, ≥2 but ≤ 7 at SCQF level 4, or >0 but ≤4 at SCQF level 5) or high attainment (>7 at SCQF level 4, ≥5 at SCQF level 5, ≥3 at SCQF level 6, or ≥1 at SCQF level 7). Unemployment was defined as not being in employment, training, or further education 6 months after leaving school.

### Covariates

We included potential sociodemographic and maternal confounders as covariates. Pupils’ sex, ethnicity, age, and postal code of residence are recorded annually by schools for the Pupil Census. Area socioeconomic status was derived from postal code of residence using the Scottish Index of Multiple Deprivation (SIMD) 2012, which is derived from 38 indicators across 7 domains (income, employment, health, housing, geographic access, crime and education, and skills and training) using information collected for data zones of residence (median population 769). Children were allocated to general population quintiles. Maternal age at delivery, parity, maternal smoking, gestational age at delivery, mode of delivery, birth weight, and 5-minute Apgar score were obtained from retrospective linkage to the SMR02 maternity database, and birth weight, sex, and gestation were combined to derive sex-gestation–specific birth weight centiles. For the purposes of employing sensitivity analyses, described in the next section, we also identified children receiving medications for diabetes, asthma, epilepsy, and skin disorders, as well as children with additional support needs attributed to the following: learning difficulty (e.g., dyslexia, dyscalculia); sensory impairment; physical motor impairment; communication problems; social, emotional, and behavioural difficulty; physical health problems; or mental health problems.

### Statistical analyses

Pupils were categorised in 2 ways. Firstly, we categorised children according to the number of eligible conditions they had, irrespective of the actual condition (0, 1, or ≥2). The characteristics of children with 1 condition and 2 or more conditions were compared to children with no conditions using chi-squared tests for trend or Spearman rank correlations for ordinal data. Outcomes experienced by children with 1 condition and 2 or more conditions were then compared against children with no conditions using univariate and multivariate models. Secondly, we categorised children according to their specific combination of conditions. The outcomes experienced by each category were then compared using univariate and multivariate models, again using children with no eligible conditions as the referent group. All multivariate models were adjusted for potential sociodemographic and maternity confounders.

Unemployment and overall attainment were analysed using binary logistic regression models. Annual number of exclusions and annual number of days absent were analysed using generalised estimating equations (GEEs) [38] with a negative binomial distribution and log link function in order to take account of correlations between repeated measurements on the same pupil across different school years. Different correlation structures were compared using the quasi-likelihood under the independence model criterion (QIC) statistic [39], with the lowest QIC indicating the most appropriate structure. The exposure time for each pupil was adjusted using the total number of possible attendances recorded each year. For all outcomes, we tested for statistical interactions with sex, socioeconomic deprivation, and age, and—where these were significant—subgroup analyses were undertaken.

We explored whether absenteeism and exclusion were potential mediators of the association between multimorbidity and academic attainment by rerunning the attainment models including each in turn as an additional covariate. We then performed formal mediation analyses using the Stata user written “paramed” command to decompose the total effect of having 2 or more conditions on attainment into direct effects (not mediated through absenteeism/exclusion) and indirect effects (mediated through absenteeism/exclusion) adjusted for sociodemographic and maternity factors. We used the same approach to explore whether attainment was a potential mediator for unemployment. We reran the unemployment model including attainment as a covariate and then performed formal mediation analyses to decompose the total effect of having 2 or more conditions on unemployment into a direct effect (not mediated through attainment) and an indirect effect (mediated through attainment) adjusted for sociodemographic and maternity factors.

Finally, we carried out brief sensitivity analyses to investigate whether the main effects of having 1 and having 2 or more conditions on each of the outcomes could be partially attributed to the associated increased risk of these affected children also having comorbid diabetes, asthma, epilepsy, or skin disorders, or additional support needs attributed to learning difficulty (e.g., dyslexia, dyscalculia); sensory impairment; physical motor impairment; communication problems; social, emotional, and behavioural difficulty; physical health problems; or mental health problems. We therefore reran the models investigating absenteeism, exclusion, attainment, and unemployment and additionally adjusted them for these comorbid conditions and any occurrence of additional support needs. All statistical analyses were conducted using Stata MP version 14.1 (StataCorp, College Station, TX).

### Approvals

Approval to undertake the study was granted by the NHS Scotland Public Benefit and Privacy Panel. Use of the health and education data was permitted through data processing and sharing agreements between the University of Glasgow, ISD, and ScotXed, respectively. The linked data extract was anonymised, then stored and analysed within the national safe haven.

### Ethics

The NHS West of Scotland Research Ethics Service confirmed that formal NHS ethics approval was not required since the study involved anonymised extracts of routinely collected data with an acceptably negligible risk of identification.

## Results

The study cohort comprised 2,793,185 records pertaining to 766,244 schoolchildren (S1 Fig), of whom 35,873 (4.7%) had at least 1 of the conditions of interest and 4,789 (0.6%) had 2 or more conditions (Table 1). Multimorbidity was more common among boys, and the prevalence increased with increasing deprivation. Children with multimorbidity had lower Apgar scores, lower gestational ages, and lower birth weight centiles and were more likely to be born via emergency cesarean section. They were more likely to be older and their mothers were more likely to be younger, have smoked during pregnancy, and be multiparous. They were also more likely to have comorbid diabetes, asthma, epilepsy, and skin disorders, as well as additional support needs attributed to learning difficulty (e.g. dyslexia, dyscalculia); sensory impairment; physical motor impairment; communication problems; social, emotional, and behavioural difficulty; physical health problems; and mental health problems. (Table 1).

**Table 1:** Sociodemographic characteristics of schoolchildren with no, 1, and 2 or more neurodevelopmental conditions.

	No conditions		1 condition		2 or more conditions		
	*N* = 725,582		*N* = 35,873		*N* = 4,789		
	*n*	Col. %	*n*	Col. %	*n*	Col. %	*P* value
***Sociodemographic factors***							
**Sex**							
Male	362,825	50.0	23,565	65.7	3,900	81.4	<0.001
Female	362,757	50.0	12,308	34.3	889	18.6	
Missing	0		0		0		
**Average age across all school years**							
Mean (SD)	10.87	4.0	11.39	3.5	11.42	3.5	<0.001
**Deprivation quintile**							
1 (most deprived)	161,875	22.3	10,590	29.6	1,329	27.8	<0.001
2	144,752	20.0	7,744	21.6	1,073	22.4	
3	140,265	19.3	6,774	18.9	885	18.5	
4	142,668	19.7	6,023	16.8	832	17.4	
5 (least deprived)	135,470	18.7	4,702	13.1	668	14.0	
Missing	552		40		2		
**Ethnic group**							
White	686,687	96.2	34,227	97.1	4,543	96.6	<0.001
Asian	16,996	2.4	630	1.8	84	1.8	
Black	1,844	0.3	68	0.2	22	0.5	
Mixed	6,408	0.9	244	0.7	35	0.7	
Other	1,879	0.3	93	0.3	17	0.4	
Missing	11,768		611		88		
***Maternity factors***							
**Maternal age (years)**							
≤24	196,112	27.0	12,192	34.0	1,574	32.9	<0.001
25–29	213,120	29.4	10,074	28.1	1,346	28.1	
30–34	207,151	28.6	8,609	24.0	1,175	24.5	
≥35	109,187	15.1	4,998	13.9	694	14.5	
Missing	12		0		0		
**Maternal smoking**							
No	468,773	72.9	19,636	61.4	2,705	63.4	<0.001
Yes	173,892	27.1	12,333	38.6	1,564	36.6	
Missing	82,917		3,904		520		
**Parity**							
0	328,313	45.5	15,223	42.7	2,129	44.8	<0.001
1	250,963	34.8	11,595	32.5	1,584	33.3	
>1	142,675	19.8	8,850	24.8	1,045	22.0	
Missing	3,631		205		31		
**Mode of delivery**							
SVD	489,228	67.4	23,902	66.6	3,089	64.5	<0.001
Assisted vaginal	87,193	12.0	3,914	10.9	550	11.5	
Breech vaginal	2,049	0.3	162	0.5	22	0.5	
Elective CS	55,167	7.6	2,770	7.7	377	7.9	
Emergency CS	91,784	12.7	5,121	14.3	751	15.7	
Missing	161		4		0		
**Sex-gestation–specific birth weight centile**							
1–3	29,020	4.0	2,187	6.1	279	5.8	<0.001
4–10	64,376	8.9	3,793	10.6	477	10.0	
11–20	86,270	11.9	4,521	12.6	557	11.6	
21–80	427,584	59.0	19,869	55.5	2,668	55.8	
81–90	62,098	8.6	2,846	7.9	419	8.8	
91–97	39,158	5.4	1,794	5.0	269	5.6	
98–100	16,152	2.2	812	2.3	115	2.4	
Missing	924		51		5		
**Gestation (weeks)**							
<28	905	0.1	210	0.6	39	0.8	<0.001
28–32	6,294	0.9	665	1.9	99	2.1	
33–36	32,861	4.5	2,410	6.7	331	6.9	
37	35,163	4.9	2,138	6.0	318	6.6	
38	90,255	12.5	5,084	14.2	653	13.6	
39	150,549	20.8	7,200	20.1	991	20.7	
40	219,360	30.3	9,805	27.4	1,265	26.4	
41	163,169	22.5	7,098	19.8	926	19.4	
42	25,767	3.6	1,198	3.3	159	3.3	
>42	728	0.1	37	0.1	5	0.1	
Missing	531		28		3		
**5-minute Apgar score**							
1–3	3,340	0.5	331	0.9	38	0.8	<0.001
4–7	6,669	0.9	546	1.5	87	1.8	
7–10	708,247	98.6	34,559	97.5	4,606	97.4	
Missing	7,326		437		58		
***Comorbid physical conditions***							
**Diabetes**							
No	722,472	99.6	35,676	99.5	4,766	99.5	<0.001
Yes	3,110	0.4	197	0.5	23	0.5	
Missing	0		0		0		
**Asthma**							
No	683,082	94.1	32,881	91.7	4,381	91.5	0.003
Yes	42,500	5.9	2,992	8.3	408	8.5	
Missing	0		0		0		
**Epilepsy**							
No	722,362	99.6	34,129	95.1	4,439	92.7	<0.001
Yes	3,220	0.4	1,744	4.9	350	7.3	
Missing	0		0		0		
**Skin disorder**							
No	603,659	83.2	28,786	80.2	3,712	77.5	<0.001
Yes	121,923	16.8	7,087	19.8	1,077	22.5	
Missing	0		0		0		
***ASNs***							
**Learning difficulty**							
No	678,866	93.6	27,598	76.9	3,492	72.9	<0.001
Yes	46,716	6.4	8,275	23.1	1,297	27.1	
Missing	0		0		0		
**Sensory impairment**							
No	721,438	99.4	34,158	95.2	4,479	93.5	<0.001
Yes	4,144	0.6	1,715	4.8	310	6.5	
Missing	0		0		0		
**Physical motor impairment**							
No	720,772	99.3	32,997	92.0	4,199	87.7	<0.001
Yes	4,810	0.7	2,876	8.0	590	12.3	
Missing	0		0		0		
**Communication problems**							
No	715,382	98.6	31,052	86.6	3,325	69.4	<0.001
Yes	10,200	1.4	4,821	13.4	1,464	30.6	
Missing	0		0		0		
**Social, emotional, or behavioural difficulty**							
No	701,020	96.6	27,850	77.6	2,600	54.3	<0.001
Yes	24,562	3.4	8,023	22.4	2,189	45.7	
Missing	0		0		0		
**Physical health condition**							
No	719,242	99.1	33,506	93.4	4,319	90.2	<0.001
Yes	6,340	0.9	2,367	6.6	470	9.8	
Missing	0		0		0		
**Mental health condition**							
No	724,728	99.9	35,038	97.7	4,393	91.7	<0.001
Yes	854	0.1	835	2.3	396	8.3	
Missing	0		0		0		
**Any ASN**							
No	644,319	88.8	18,068	50.4	1,328	27.7	<0.001
Yes	81,263	11.2	17,805	49.6	3,461	72.3	
Missing	0		0		0		

*P* values obtained using chi-squared tests for trend, Spearman rank correlation coefficients, or analysis of variance.

“Any ASN” includes those attributed to learning difficulty; sensory impairment; physical motor impairment; communication problems; social, emotional, and behavioural difficulty; physical health problems; or mental health problems.

**Abbreviations:** ASN, additional support need; Col. %, column percentage; CS, cesarean section; SD, standard deviation; SVD, spontaneous vaginal delivery

The percentage of data missing within each variable was less than 0.2% excluding parity (0.6%), Apgar score (1.1%), ethnicity (1.8%), and smoking during pregnancy (9.9%) (Table 1). Missing values for the latter 2 were analysed as “unknown.” Of the 10,814 children who had ASD, 3,574 (33.0%) had at least 1 other condition. Of the 7,413 with ADHD, 2,164 (29.2%) had at least 1 comorbidity. Of the 22,292 with intellectual disabilities, 3,685 (16.5%) had comorbidity, and of the 5,342 with depression, 565 (10.6%) had comorbidity. The most common combination was ASD with intellectual disabilities, which occurred in 2,251 (0.3%) children; 1,823 (81.0%) children with this combination were boys (Table 2).

**Table 2:** Number and percentage of children with each combination of condition overall and by sex, age, and deprivation quintile.

	Overall		Boys		Girls		SIMD1 most deprived		SIMD2		SIMD3		SIMD4		SIMD5 least deprived		Missing SIMD	Average age across all school years	
	*N =* 766,244		*N =* 390,290		*N =* 375,954		*N =* 173,794		*N =* 153,569		*N =* 147,924		*N =* 149,523		*N =* 140,840		*N =* 594	*N =* 766,244	
	*n*	%	*n*	%	*n*	%	*n*	%	*n*	%	*n*	%	*n*	%	*n*	%	*n*	Mean	SD
**No conditions**	725,582	94.7	362,825	93.0	362,757	96.5	161,875	93.1	144,752	94.3	140,265	94.8	142,668	95.4	135,470	96.2	552	10.87	3.99
**ASD only**	7,240	0.9	6,226	1.6	1,014	0.3	1,799	1.0	1,453	0.9	1,423	1.0	1,382	0.9	1,178	0.8	5	10.86	3.59
**ADHD only**	5,249	0.7	4,443	1.1	806	0.2	1,756	1.0	1,296	0.8	962	0.7	740	0.5	492	0.3	3	11.67	3.2
**Depression only**	4,777	0.6	1,380	0.4	3,397	0.9	902	0.5	967	0.6	1,029	0.7	965	0.6	906	0.6	8	14.51	2.13
**ID only**	18,607	2.4	11,516	3.0	7,091	1.9	6,133	3.5	4,028	2.6	3,360	2.3	2,936	2.0	2,126	1.5	24	10.72	3.48
**ASD + ADHD**	771	0.1	704	0.2	67	0.0	204	0.1	166	0.1	149	0.1	138	0.1	114	0.1	0	11.22	3.12
**ASD + depression**	180	0.0	133	0.0	47	0.0	26	0.0	39	0.0	45	0.0	41	0.0	29	0.0	0	13.57	2.49
**ADHD + depression**	109	0.0	77	0.0	32	0.0	32	0.0	30	0.0	20	0.0	16	0.0	11	0.0	0	13.66	2.11
**ID + ASD**	2,251	0.3	1,823	0.5	428	0.1	622	0.4	479	0.3	422	0.3	382	0.3	346	0.2	0	10.91	3.74
**ID + ADHD**	939	0.1	772	0.2	167	0.0	321	0.2	230	0.1	157	0.1	143	0.1	87	0.1	1	11.60	3.09
**ID + depression**	147	0.0	65	0.0	82	0.0	36	0.0	38	0.0	30	0.0	24	0.0	19	0.0	0	13.62	2.62
Three or more conditions*	392	0.1	326	0.1	66	0.0	88	0.1	91	0.1	62	0.0	88	0.1	62	0.0	1	11.93	3.07

*Unable to provide numbers for specific combinations due to small numbers.

**Abbreviations:** ADHD, attention deficit hyperactivity disorder; ASD autism spectrum disorder; ID, intellectual disability; SIMD, Scottish Index of Multiple Deprivation

Multimorbidity was associated with significantly increased risk of school absenteeism, exclusion from school, low attainment, and subsequent unemployment with statistically significant dose relationships evident between the number of neurodevelopmental conditions (0, 1, ≥2) and the last 3 outcomes (Table 3). Analyses of absenteeism and exclusion included 1,597,397 records for 702,210 children. Compared to children with none of the conditions (median 7.5 days), children with 1 condition and children with 2 or more conditions had more absences per year (medians 10 and 8.5 days, respectively). Compared to children with no conditions, children with 1 condition and children with 2 or more conditions had more absenteeism on univariate analysis (1 condition incidence rate ratio [IRR] 1.38, 95% CI 1.36–1.39, *p <* 0.001 and 2 or more conditions IRR 1.25, 95% CI 1.21–1.29, *p <* 0.001) and after adjustment for sociodemographic and maternity confounders (1 condition adjusted IRR 1.28, 95% CI 1.27–1.30, *p <* 0.001 and 2 or more conditions adjusted IRR 1.23, 95% CI 1.20–1.28, *p <* 0.001). There were significant interactions with sex, age, and deprivation (all *p <* 0.001) whereby the magnitude of the association between multimorbidity and absenteeism was greater in girls than boys (adjusted IRR 1.35, 95% CI 1.25–1.45, *p <* 0.001 versus adjusted IRR 1.20, 95% CI 1.16–1.25, *p <* 0.001), was stronger among children younger than 11 years of age (adjusted IRR 1.26, 95% CI 1.21–1.32, *p <* 0.001) compared to children older than 14 years of age (adjusted IRR 1.12, 95% CI 1.05–1.20, *p <* 0.01), and was stronger among children in the least compared to the most deprived quintile (adjusted IRR 1.38, 95% CI 1.25–1.51, *p <* 0.001 versus adjusted IRR 1.06, 95% CI 1.00–1.13, *p* = 0.052) (Table 3). However, this latter finding was explained by absenteeism already being higher in more deprived quintiles among children with no morbidity; median 11.0 days versus median 5.0 days in the most and least deprived quintiles, respectively. Among children with multimorbidity, absenteeism remained more common in the most deprived quintile compared with the least; median 10.8 days versus median 6.0 days, respectively (S1 Table).

**Table 3:** Multivariate associations between number of neurodevelopmental conditions and education outcomes: Overall and by sex, age, and deprivation quintile.

		Absence^a^		Exclusion^a^		Low attainment^b^		Unemployment^c^	
		Univariate model	Multivariate model^d^	Univariate model	Multivariate model^d^	Univariate model	Multivariate model^d^	Univariate model	Multivariate model^d^
	Number of conditions	IRR (95% CI)		IRR (95% CI)		OR (95% CI)		OR (95% CI)	
**Overall**	None	1.00	1.00	1.00	1.00	1.00	1.00	1.00	1.00
	One	1.38 (1.36–1.39)	1.28 (1.27–1.30)	3.37 (3.21–3.54)	2.37 (2.25–2.48)	2.52 (2.39–2.66)	3.92 (3.63–4.23)	2.00 (1.90–2.11)	1.57 (1.49–1.66)
	Two or more	1.25 (1.21–1.29)	1.23 (1.20–1.28)	4.55 (4.10–5.05)	3.04 (2.74–3.38)	5.03 (4.10–6.16)	12.07 (9.15–15.94)	2.60 (2.26–2.98)	2.11 (1.83–2.45)
**Boys**	None	1.00	1.00	1.00	1.00	1.00	1.00	1.00	1.00
	One	1.31 (1.29–1.33)	1.22 (1.20–1.24)	2.90 (2.75–3.06)	2.41 (2.29–2.55)	3.02 (2.80–3.25)	4.51 (4.06–5.01)	1.89 (1.77–2.02)	1.50 (1.40–1.60)
	Two or more	1.22 (1.17–1.26)	1.20 (1.16–1.25)	3.24 (2.90–3.62)	2.87 (2.57–3.22)	4.26 (3.38–5.35)	11.66 (8.51–15.97)	2.29 (1.96–2.67)	2.05 (1.74–2.41)
**Girls**	None	1.00	1.00	1.00	1.00	1.00	1.00	1.00	1.00
	One	1.52 (1.49–1.55)	1.39 (1.36–1.41)	2.85 (2.54–3.20)	2.07 (1.84–2.32)	1.98 (1.83–2.14)	3.36 (3.01–3.77)	2.06 (1.89–2.24)	1.77 (1.62–1.93)
	Two or more	1.45 (1.34–1.56)	1.35 (1.25–1.45)	6.01 (4.45–8.13)	4.34 (3.23–5.83)	5.87 (3.80–9.08)	12.57 (7.05–22.38)	2.86 (2.11–3.89)	2.34 (1.70–3.24)
**Age <11 years**	None	1.00	1.00	1.00	1.00		N/A		N/A
	One	1.35 (1.33–1.37)	1.23 (1.21–1.25)	7.69 (6.93–8.53)	4.42 (3.96–4.93)		N/A		N/A
	Two or more	1.33 (1.28–1.38)	1.26 (1.21–1.32)	12.01 (9.82–14.69)	6.51 (5.28–8.03)		N/A		N/A
**Age 11–14 years**	None	1.00	1.00	1.00	1.00		N/A		N/A
	One	1.40 (1.38–1.43)	1.31 (1.28–1.33)	3.07 (2.87–3.28)	2.18 (2.04–2.33)		N/A		N/A
	Two or more	1.30 (1.24–1.37)	1.27 (1.21–1.34)	3.57 (3.08–4.15)	2.47 (2.12–2.87)		N/A		N/A
**Age >14 years**	None	1.00	1.00	1.00	1.00		N/A		N/A
	One	1.39 (1.37–1.42)	1.36 (1.33–1.39)	2.37 (2.19–2.55)	1.77 (1.64–1.90)		N/A		N/A
	Two or more	1.11 (1.04–1.18)^f^	1.12 (1.05–1.20)^f^	3.38 (2.85–4.00)	2.37 (1.98–2.82)		N/A		N/A
**SIMD cat. 1**^e^	None	1.00	1.00	1.00	1.00	1.00	1.00	1.00	1.00
	One	1.17 (1.15–1.20)	1.16 (1.14–1.18)	2.47 (2.30–2.66)	2.04 (1.89–2.19)	2.25 (2.00–2.54)	3.22 (2.74–3.78)	1.55 (1.41–1.69)	1.42 (1.29–1.56)
	Two or more	1.01 (0.95–1.07)^g^	1.06 (1.00–1.13)^h^	2.84 (2.39–3.36)	2.11 (1.77–2.51)	3.70 (2.25–6.09)	5.11 (2.67–9.76)	1.17 (0.87–1.57)^i^	1.09 (0.81–1.48)^j^
**SIMD cat. 2**	None	1.00	1.00	1.00	1.00	1.00	1.00	1.00	1.00
	One	1.28 (1.25–1.31)	1.24 (1.21–1.27)	3.10 (2.82–3.40)	2.36 (2.15–2.59)	2.27 (2.02–2.55)	3.35 (2.84–3.94)	1.69 (1.52–1.89)	1.44 (1.29–1.61)
	Two or more	1.19 (1.11–1.27)	1.17 (1.10–1.24)	4.31 (3.51–5.29)	2.87 (2.35–3.52)	5.71 (3.62–8.98)	21.58 (12.08–38.56)	2.63 (2.01–3.45)	2.41 (1.82–3.19)
**SIMD cat. 3**	None	1.00	1.00	1.00	1.00	1.00	1.00	1.00	1.00
	One	1.41 (1.37–1.45)	1.33 (1.30–1.37)	3.83 (3.40–4.31)	2.77 (2.46–3.11)	2.64 (2.35–2.96)	4.06 (3.46–4.77)	1.95 (1.72–2.20)	1.60 (1.41–1.81)
	Two or more	1.37 (1.28–1.47)	1.35 (1.25–1.45)	5.72 (4.49–7.28)	3.94 (3.08–5.04)	4.38 (2.88–6.66)	11.01 (6.33–19.16)	2.80 (2.05–3.82)	2.46 (1.77–3.40)
**SIMD cat. 4**	None	1.00	1.00	1.00	1.00	1.00	1.00	1.00	1.00
	One	1.43 (1.39–1.47)	1.36 (1.32–1.40)	3.84 (3.31–4.44)	2.79 (2.40–3.24)	2.98 (2.63–3.36)	5.37 (4.50–6.40)	2.62 (2.28–3.00)	2.21 (1.92–2.55)
	Two or more	1.38 (1.27–1.49)	1.35 (1.25–1.45)	8.07 (6.14–10.60)	5.19 (3.91–6.88)	5.91 (3.75–9.33)	12.64 (6.95–23.02)	4.97 (3.58–6.88)	4.06 (2.89–5.70)
**SIMD cat. 5**^e^	None	1.00	1.00	1.00	1.00	1.00	1.00	1.00	1.00
	One	1.57 (1.52–1.63)	1.46 (1.41–1.51)	4.91 (4.05–5.94)	3.26 (2.69–3.95)	2.87 (2.49–3.31)	4.17 (3.41–5.11)	2.96 (2.51–3.49)	2.47 (2.08–2.93)
	Two or more	1.40 (1.27–1.54)	1.38 (1.25–1.51)	7.71 (5.39–11.01)	4.57 (3.16–6.63)	7.58 (4.59–12.54)	13.65 (6.40–29.13)	4.78 (3.24–7.04)	4.17 (2.79–6.23)

^a^1,597,397 records (702,018 pupils) analysed using GEEs with a negative binomial distribution and log link function to produce IRRs.

^b^139,205 pupils analysed using binary logistic regression to produce ORs.

^c^217,924 pupils analysed using binary logistic regression to produce ORs.

^d^All multivariate models adjusted for age, sex, deprivation quintile, ethnic group, maternal age, maternal smoking, parity, mode of delivery, gestation at delivery, sex-gestation–specific birth weight centile, and 5-minute Apgar score.

^e^SIMD cat. 1 = most deprived; SIMD cat 5 = least deprived.

^f^*p <* 0.01.

^g^*p* = 0.799.

^h^*p* = 0.052.

^i^*p* = 0.299.

^j^*p* = 0.570.

All models significant at *p <* 0.001 except for those indicated with a footnote. “N/A” indicates that subgroup analyses didn’t apply as all children in these analyses were aged 14 years or older.

**Abbreviations:** cat., category; GEE, generalised estimating equation; IRR, incidence rate ratio; OR, odds ratio; SIMD, Scottish Index of Multiple Deprivation

Of children with 2 or more conditions, 12.8% (589/4,589) were excluded from school at least once during the study period compared to 9.8% (3,362/34,463) of children with 1 condition and 3.5% (22,966/663,158) of children with no conditions. Compared to children with no conditions, children with 1 condition and children with 2 or more conditions had more exclusion on univariate analysis (1 condition IRR 3.37, 95% CI 3.21–3.54, *p <* 0.001 and 2 or more conditions IRR 4.55, 95% CI 4.10–5.05, *p <* 0.001) and after adjustment for sociodemographic and maternity confounders (1 condition adjusted IRR 2.37, 95% CI 2.25–2.48, *p <* 0.001 and 2 or more conditions adjusted IRR 3.04, 95% CI 2.74–3.38, *p <* 0.001). There were significant interactions with sex, age, and deprivation (all *p <* 0.001) whereby the magnitude of the association between multimorbidity and exclusion was greater in girls than boys (adjusted IRR 4.34, 95% CI 3.23–5.83, *p <* 0.001 versus adjusted IRR 2.87, 95% CI 2.57–3.22, *p <* 0.001), was stronger among children younger than 11 years of age (adjusted IRR 6.51, 95% CI 5.28–8.03, *p <* 0.001) compared to children older than 14 years of age (adjusted IRR 2.37, 95% CI 1.98–2.82, *p <* 0.001), and was stronger among children in the least compared to the most deprived quintile (adjusted IRR 4.57, 95% CI 3.16–6.63, *p <* 0.001 versus adjusted IRR 2.11, 95% CI 1.77–2.51, *p <* 0.001) (Table 3). However, this latter finding was explained by exclusion already being higher in more deprived quintiles among children with no morbidity. The percentage of children with no morbidity who experienced school exclusion was 6.6% (9,734/148,478) in the most deprived quintile compared with 1.2% (1,462/123,533) in the least deprived. For children with multimorbidity, the corresponding figures were 15.9% (202/1,269) and 7.8% (49/625), respectively (S1 Table).

On analysing exam grades for 139,205 children, 476 (0.3%) had 2 or more conditions. The percentage obtaining low academic attainment was higher among children with 2 or more conditions (73.1%, 348/476) compared to children with 1 condition (57.7%, 3,436/5,953) and children with no conditions (35.1%, 46,607/132,776). Compared to children with no conditions, children with 1 condition and children with 2 or more conditions had poorer attainment on univariate analysis (1 condition odds ratio [OR] 2.52, 95% CI 2.39–2.66, *p <* 0.001 and 2 or more conditions OR 5.03, 95% CI 4.10–6.16, *p <* 0.001) and after adjustment for sociodemographic and maternity confounders (1 condition adjusted OR 3.92, 95% CI 3.63–4.23, *p <* 0.001 and 2 or more conditions adjusted OR 12.07, 95% CI 9.15–15.94, *p <* 0.001). There were significant interactions with sex (*p <* 0.001) and deprivation (*p <* 0.001) whereby the magnitude of the association between multimorbidity and attainment was greater in girls than boys (adjusted OR 12.57, 95% CI 7.05–22.38, *p <* 0.001 versus adjusted OR 11.66, 95% CI 8.51–15.97, *p <* 0.001) and stronger among children in the least compared to the most deprived quintile (adjusted OR 13.65, 95% CI 6.40–29.13, *p <* 0.001 versus adjusted OR 5.11, 95% CI 2.67–9.76, *p <* 0.001) (Table 3). However, this latter finding was explained by poorer attainment being more common in more deprived quintiles among children with no morbidity. The percentage of children with no morbidity who had poor academic attainment was 54.7% (15,682/28,658) in the most deprived quintile compared with 15.8% (3,939/24,932) in the least deprived. For children with multimorbidity, the corresponding figures were 81.7% (85/104) and 58.7% (37/63), respectively (S1 Table). Inclusion of days absent as a covariate attenuated the strength of the association between multimorbidity and low attainment, but the association remained significant (adjusted OR 11.32, 95% CI 8.48–15.10, *p <* 0.001). A formal mediation analysis confirmed that the effect of multimorbidity on attainment (adjusted OR 13.84, 95% CI 8.92–18.55, *p <* 0.001) was likely to be a direct effect (adjusted OR 13.50, 95% CI 8.66–18.47, *p <* 0.001) rather than an indirect effect mediated by absenteeism (adjusted OR 1.02, 95% CI 0.98–1.08, *p* = 0.643). Inclusion of exclusions as a covariate also attenuated the strength of the association between multimorbidity and low attainment, but again the association remained significant (adjusted OR 10.69, 95% CI 8.04–14.20, *p <* 0.001). A formal mediation analysis suggested that most of the effect of multimorbidity on attainment (adjusted OR 14.08, 95% CI 9.00–19.65, *p <* 0.001) was a direct effect (adjusted OR 11.14, 95% CI 7.33–15.47, *p <* 0.001) with a small portion attributed to an indirect effect mediated by exclusion (adjusted OR 1.26, 95% CI 1.20–1.36, *p <* 0.001).

Of 217,924 children leaving school over the study period, 1,164 (0.5%) had 2 or more conditions. Unemployment was higher among children with 2 or more conditions (22.3%, 260/1,164) compared to children with 1 condition (18.1%, 1,874/10,333) and children with no conditions (10.0%, 20,585/206,427). Compared to children with no conditions, children with 1 condition and children with 2 or more conditions had increased unemployment on univariate analysis (1 condition OR 2.00, 95% CI 1.90–2.11, *p <* 0.001 and 2 or more conditions OR 2.60, 95% CI 2.26–2.98, *p <* 0.001) and after adjustment for sociodemographic and maternity confounders (1 condition adjusted OR 1.57, 95% CI 1.49–1.66, *p <* 0.001 and 2 or more conditions adjusted OR 2.11, 95% CI 1.83–2.45, *p <* 0.001). There was a significant interaction with deprivation (*p <* 0.001) whereby there was a significant association between multimorbidity and unemployment among children in the least deprived quintile but not for children in the most deprived quintile (adjusted OR 4.17, 95% CI 2.79–6.23, *p <* 0.001 versus adjusted OR 1.09, 95% CI 0.81–1.48, *p* = 0.570) (Table 3). However, this was explained by unemployment being higher in more deprived quintiles among children with no morbidity. The percentage of children with no morbidity who were unemployed 6 months after leaving school was 16.5% (7,431/45,009) in the most deprived quintile compared with 4.7% (1,831/39,269) in the least deprived. For children with multimorbidity, the corresponding figures were 18.8% (55/293) and 18.9% (32/169), respectively (S1 Table). Following inclusion of attainment as a covariate, the association between multimorbidity and unemployment was no longer statistically significant (adjusted OR 1.13, 95% CI 0.87–1.48, *p* = 0.368). A formal mediation analysis confirmed that the effect of multimorbidity on unemployment (adjusted OR 2.16, 95% CI 1.56–2.78, *p <* 0.001) was likely to be an indirect effect mediated by attainment (adjusted OR 1.91, 95% CI 1.77–2.02, *p <* 0.001) rather than a direct effect (adjusted OR 1.13, 95% CI 0.84–1.48, *p* = 0.359).

On sensitivity analyses, the main associations between multimorbidity and each of the outcomes (absences, exclusions, attainment, and unemployment) remained, and the effect sizes were of similar magnitude as before. This suggested that the associations were irrespective of the increased physical comorbidities and additional support needs experienced by these affected children (Table 4).

**Table 4:** Multivariate associations between number of neurodevelopmental conditions and education outcomes: Sensitivity analyses adjusting for physical comorbidities and additional support needs.

		Absence^a^	Exclusion^a^	Low attainment^b^	Unemployment^c^
	Number of conditions	IRR (95% CI)	IRR (95% CI)	OR (95% CI)	OR (95% CI)
**Adjusted for sociodemographic and maternity factors**^d^	None	1.00	1.00	1.00	1.00
	One	1.28 (1.27–1.30)	2.37 (2.25–2.48)	3.92 (3.63–4.23)	1.57 (1.49–1.66)
	Two or more	1.23 (1.20–1.28)	3.04 (2.74–3.38)	12.07 (9.15–15.94)	2.11 (1.83–2.45)
**Adjusted for sociodemographic and maternity factors**^d^ **and comorbid physical conditions**^e^	None	1.00	1.00	1.00	1.00
	One	1.26 (1.25–1.28)	2.40 (2.28–2.52)	3.80 (3.52–4.10)	1.54 (1.46–1.63)
	Two or more	1.21 (1.17–1.25)	3.12 (2.81–3.47)	11.80 (8.94–15.58)	2.04 (1.76–2.36)
**Adjusted for sociodemographic and maternity factors**^d^**, comorbid physical conditions**^e^**, and any record of additional support needs**^f^	None	1.00	1.00	1.00	1.00
	One	1.22 (1.21–1.23)	1.83 (1.74–1.93)	3.16 (2.92–3.42)	1.48 (1.40–1.57)
	Two or more	1.13 (1.09–1.17)	2.05 (1.84–2.28)	7.42 (5.58–9.86)	1.89 (1.63–2.19)

^a^1,597,397 records (702,018 pupils) analysed using GEEs with a negative binomial distribution and log link function to produce IRRs.

^b^139,205 pupils analysed using binary logistic regression to produce ORs.

^c^217,924 pupils analysed using binary logistic regression to produce ORs.

^d^Adjusted for age, sex, deprivation quintile, ethnic group, maternal age, maternal smoking, parity, mode of delivery, gestation at delivery, sex- gestation–specific birth weight centile, and 5-minute Apgar score.

^e^Adjusted for diabetes, asthma, epilepsy, and skin disorders.

^f^Adjusted for any additional support needs attributable to learning difficulty, sensory impairment, physical motor impairment, communication problems, social, emotional, and behavioural difficulty, physical health problems, and mental health problems.

All models significant at *p <* 0.001.

**Abbreviations:** GEE, generalised estimating equation; IRR, incidence rate ratio; OR, odds ratio

**Fig 1:**
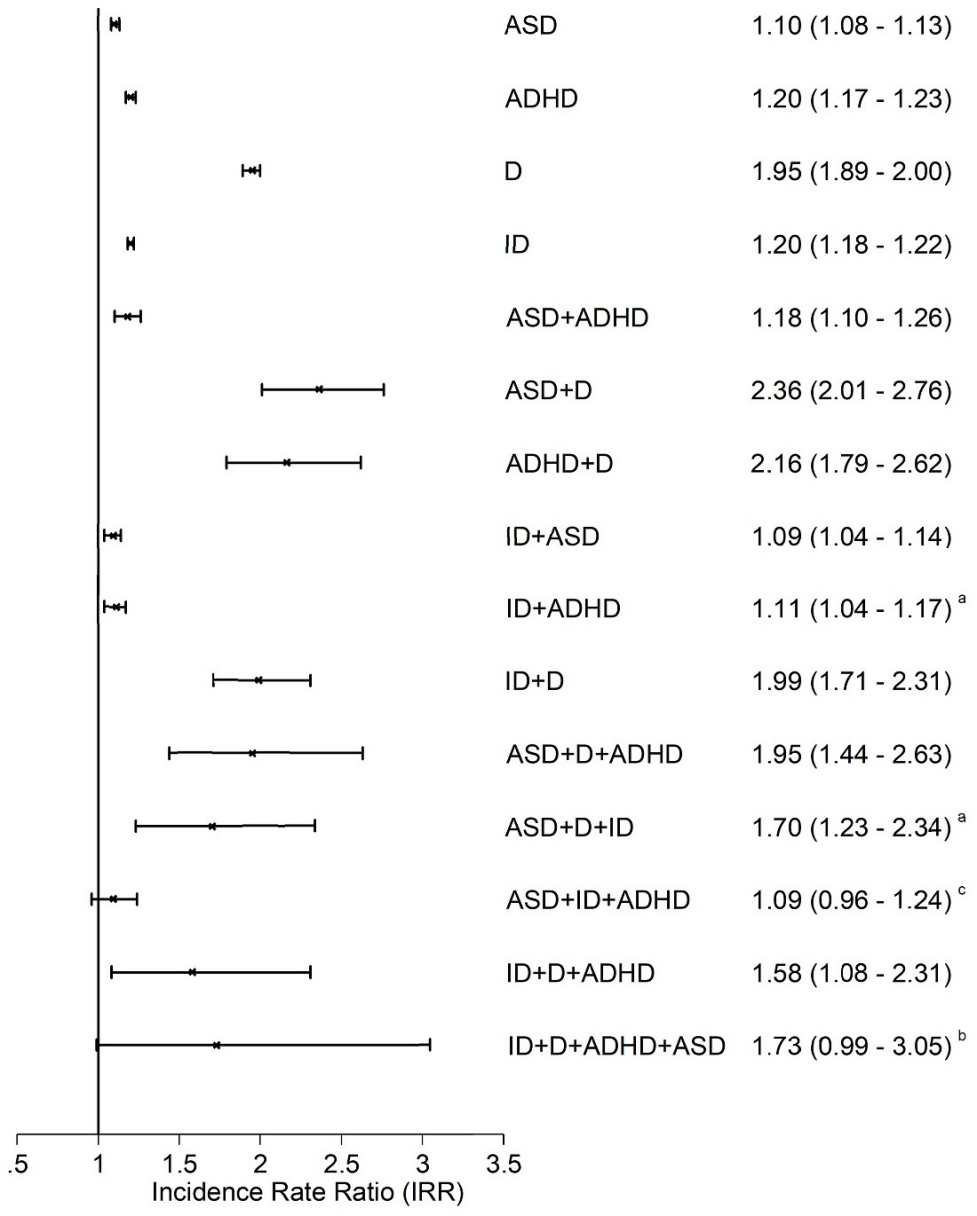
Associations between different combinations of neurodevelopmental conditions and absenteeism. Adjusted for age, sex, deprivation quintile, ethnic group, maternal age, maternal smoking, parity, mode of delivery, gestation at delivery, sex-gestation–specific birth weight centile, and 5-minute Apgar score. All categories referent to children with no conditions. 1,597,397 records (702,018 pupils) analysed using GEEs with a negative binomial distribution and log link function to produce IRRs. Bars represent 95% confidence intervals. All models significant at *p <* 0.001 except ^a^*p <* 0.01; ^b^*p* = 0.055; ^c^*p* = 0.165. ADHD, attention deficit hyperactivity disorder; ASD, autism spectrum disorder; D, depression; GEE, generalised estimating equation; ID, intellectual disability; IRR, incidence rate ratio.

**Fig 2:**
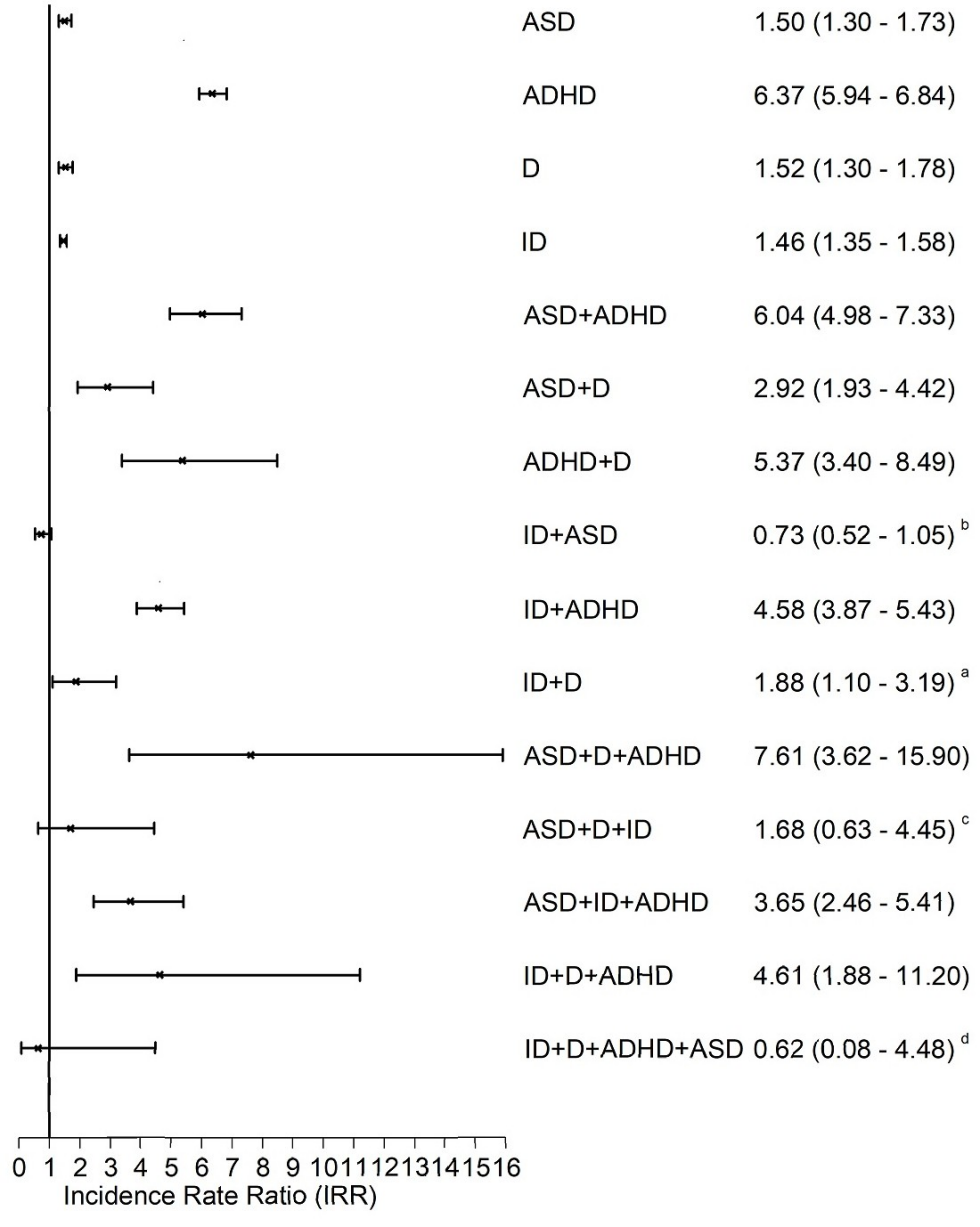
Associations between different combinations of neurodevelopmental conditions and exclusion. Adjusted for age, sex, deprivation quintile, ethnic group, maternal age, maternal smoking, parity, mode of delivery, gestation at delivery, sex-gestation–specific birth weight centile, and 5minute Apgar score. All categories referent to children with no conditions. 1,597,397 records (702,018 pupils) analysed using GEEs with a negative binomial distribution and log link function to produce IRRs. Bars represent 95% confidence intervals. All models significant at *p <* 0.001 except ^a^*p <* 0.05; ^b^*p* = 0.088; ^c^*p* = 0.300; ^d^*p* = 0.631. ADHD, attention deficit hyperactivity disorder; ASD, autism spectrum disorder; D, depression; GEE, generalised estimating equation; ID, intellectual disability; IRR, incidence rate ratio.

On analysing specific combinations of the 4 neurodevelopmental conditions of interest versus children with no conditions, we observed that the strongest driver of absenteeism was coexistence of depression. The unadjusted and adjusted IRRs were 1.01 (95% CI 0.98–1.04 [*p* = 0.529]) and 1.10 (95% CI 1.08–1.13 [*p <* 0.001]), respectively, for isolated ASD; 1.43 (95% CI 1.39–1.47 [*p <* 0.001]) and 1.20 (95% CI 1.17–1.23 [*p <* 0.001]), respectively, for isolated ADHD; and 1.28 (95% CI 1.26–1.30 [*p <* 0.001]) and 1.20 (95% CI 1.18–1.22 [*p <* 0.001]), respectively, for isolated intellectual disabilities. For children with concomitant depression, the corresponding figures were 2.30 (95% CI 1.99–2.66 [*p <* 0.001]) and 2.36 (95% CI 2.01–2.76 [*p <* 0.001]), 2.57 (95% CI 2.13–3.09 [*p <* 0.001]) and 2.16 (95% CI 1.79–2.62 [*p <* 0.001]), and 2.37 (95% CI 2.06–2.74 [*p <* 0.001]) and 1.99 (95% CI 1.71–2.31 [*p <* 0.001]), respectively (Fig 1). The strongest driver of exclusion from school was coexistence of ADHD. The unadjusted and adjusted IRRs were 1.81 (95% CI 1.57–2.08 [*p <* 0.001]) and 1.50 (95% CI 1.30–1.73 [*p <* 0.001]), respectively, for isolated ASD; 1.74 (95% CI 1.48–2.04 [*p <* 0.001]) and 1.52 (95% CI 1.30–1.78 [*p <* 0.001]), respectively, for isolated depression; and 2.07 (95% CI 1.91–2.24 [*p <* 0.001]) and 1.46 (95% CI 1.35–1.58 [*p <* 0.001]), respectively, for isolated intellectual disabilities. For children with concomitant ADHD, these increased to 7.96 (95% CI 6.59–9.60 [*p <* 0.001]) and 6.04 (95% CI 4.98–7.33 [*p <* 0.001]), 9.73 (95% CI 6.31–14.99 [*p <* 0.001]) and 5.37 (95% CI 3.40–8.49 [*p <* 0.001]), and 9.01 (95% CI 7.67–10.58 [*p <* 0.001]) and 4.58 (95% CI 3.87–5.43 [*p <* 0.001]), respectively (Fig 2). The associations with low attainment and unemployment did not appear to be driven by specific conditions; rather they displayed nonspecific dose relationships whereby increasing numbers of concomitant conditions were associated with increased risk (S2 and S3 Figs).

## Discussion

We retrospectively linked Scotland-wide education and health data to analyse outcomes among 766,244 children attending Scottish schools between 2009 and 2013. We ascertained ASD and intellectual disabilities from records of additional support needs and ADHD and depression through relevant encashed prescriptions and identified children with neurodevelopmental multimorbidity (≥2 of these conditions) before comparing them against peers. Children with multimorbidity fared worse across all the educational outcomes measured, experiencing greater absenteeism and exclusion, poorer attainment, and increased unemployment even after adjusting for sociodemographic and maternity factors, comorbid physical conditions, and additional support needs. Significant dose relationships were evident between number of conditions (0, 1, ≥2) and the last 3 outcomes. Girls were less likely to have multimorbidity but experienced greater adverse impact on educational outcomes than boys if they did. Multimorbidity most commonly took the form of coexisting ASD and intellectual disability. ADHD, on its own or coexisting with other conditions, was the strongest observed driver behind increased exclusion from school. Depression was less common, but when present, it was the strongest observed driver of school absenteeism. Low attainment and unemployment were less susceptible to specific conditions; rather they were more common among children with multimorbidity independent of the specific combination of conditions. The association between multimorbidity and attainment was a direct effect not mediated by absenteeism but partially mediated by exclusion. The association between multimorbidity and unemployment was an indirect effect mediated through poorer attainment.

Our findings fill a gap in previous research regarding neurodevelopmental multimorbidity in children. Previous studies investigating singular or comorbid neurodevelopmental conditions have used small cohorts, whereas larger studies have focussed on educational outcomes associated with single conditions, such as our own work on ADHD and depression [24, 28, 34]. To our knowledge, no previous studies in Scotland, the UK, or worldwide have investigated the associations between neurodevelopmental multimorbidity and measurable educational outcomes in an unselected, population-wide cohort. Our study was a large, nonselective study including children across the whole of Scotland. However, to enable our results to be more widely generalizable, the study findings should be corroborated in other countries where there may be differences in educational support.

Previous smaller sample studies that have investigated school outcomes of children with co-occurring conditions have mainly focussed on comorbid ADHD with respect to academic attainment using battery tests [32] or parental, teacher, or pupil feedback [31, 33, 35] and have reported poorer outcomes in these children. These studies reported outcomes of children with ADHD and comorbid ASD [31], depression [32], and learning difficulty (e.g. dyslexia, dyscalculia) [33,35]. Our findings of poorer academic attainment based on school recorded exam results agree with these studies on children with comorbid ADHD and ASD, and on children with comorbid ADHD and depression.

We observed some sex differences both in terms of prevalence of conditions, in combination or in isolation, and in terms of how these conditions affected the outcomes. Schools may have a lower awareness or experience of ASD in girls particularly given that the prevalence is 3 times higher in boys [40]. Previous research suggests that female presentation of ASD may differ from that in males and may not be as well detected by standard measures [41, 42]. ASD in females of average intelligence may be “masked” by other conditions, such as eating disorders [43], anxiety disorders, and borderline personality disorders [44]. Females with ASD may be better than males at hiding their difficulties by copying social interaction and having better language skills, different special interests, and less hyperactivity and aggression [45, 46]. While depression is more common in girls [47], affected boys are more likely to exhibit violence and misbehaviour, whereas girls are more likely to withdraw or experience less visible symptoms such as sleep problems, eating disorders, reduced motivation, and self-harm [48]. Therefore, schools may be less likely to recognise girls as needing support. Similarly, girls who have ADHD more often present with subtle symptoms such as inattention, whereas boys more commonly present with impulsivity and hyperactivity [49]. Girls are both less likely to have a referral for medical assessment and less likely to use medication [49, 50]. This may explain why girls with known ADHD have a significantly higher rate of exclusion than boys with the condition [24, 34].

Multimorbidity was more likely to result in exclusion in younger children and absenteeism in older children. Underlying disorders may be less well recognised or managed in younger pupils, and therefore disruptive behaviours more commonly result in exclusions. Another factor may be that older children have greater autonomy and less parental control and are, therefore, more able to personally choose to be absent from school. We observed that the most commonly occurring combination of conditions was intellectual disability and autism. This concurs with previous literature, and there is evidence of shared genetics [51]. Children with autism have a wide range of intellectual abilities, ranging from children with severe intellectual disabilities to those with above normal intellectual abilities. On balance, we felt it best to present children with and without intellectual disabilities separately.

We observed that children with neurodevelopmental comorbidity also had increased prevalence of physical disorders such as diabetes, asthma, epilepsy, and skin disorders, as well as increased risk of having additional support needs attributed to learning difficulty (e.g., dyslexia, dyscalculia); sensory impairment; physical motor impairment; communication problems; social, emotional, and behavioural difficulty; physical health problems; and mental health problems. The association with comorbid epilepsy is not surprising and is likely attributable to the previously reported high prevalence among people with intellectual disability [52, 53]. The increased prevalence of diabetes, asthma, and skin disorders such as psoriasis among children with neurodevelopmental disorders has also been reported elsewhere [53]. In addition to poorer educational outcomes, including increased additional support needs, among children treated for ADHD and depression, we have also previously reported poorer school outcomes among children treated for diabetes, asthma, and epilepsy [54–56]. Increased additional support needs is not surprising given that these children may require assistance due to wider comorbidities or symptoms directly related to their combination of conditions. Results from our sensitivity analyses, however, suggest that the observed associations between neurodevelopmental multimorbidity and absenteeism, exclusion, attainment, and unemployment were irrespective of the existence of comorbid physical conditions and other reasons for additional support needs.

Our study had several strengths. Our cohort of 766,244 pupils was population-wide and provided enough statistical power to test for interactions and conduct subgroup analyses where appropriate. We were able to adjust for a range of sociodemographic—as well as maternal and pregnancy related—confounders identified through linkage to maternity records, and we were able to identify comorbid physical conditions and additional support needs to enable sensitivity analyses. The 4 school outcomes share potential associations of their own. For example, repeated exclusions contribute directly to days absent from school, absences and exclusions negatively affect attainment, and school performance influences future employment opportunities. We have highlighted some of these associations previously [55]. Therefore, to account for and better explain the intricacies of these relationships, we used robust mediation modelling in order to decompose main effects into direct and indirect effects. Identification of affected children through school records and community prescriptions, rather than hospital attendance, reduced the likelihood of ascertainment being restricted to more severe cases with the exception of depression since psychological interventions rather than antidepressants are the first-line treatments in depressed children and adolescents. Missing data across all covariates did not exceed 1.9% and, for most analyses, was considerably lower. Given the sample size, we do not believe this affected the results, and therefore we did not impute data or run sensitivity analyses.

Nevertheless, there were also some limitations. Our ascertainment methods failed to include children not yet diagnosed, children diagnosed but not receiving treatment for ADHD, and children either not receiving treatment or receiving solely nonmedicinal treatments such as cognitive behavioural therapy for depression. Conversely, over-ascertainment may have occurred due to the nonspecific nature of some of the medications. In particular, we ascertained depression using encashed prescriptions for tricyclic antidepressants, SSRIs, mirtazapine, or venlafaxine. Some of these drugs have other indications such as OCD, anxiety disorders, irritability, or enuresis, and without primary care records, we could not confirm the clinical indications for medication. However, these conditions often occur in conjunction with depression, and the prevalence of enuresis was low in the age range studied. Furthermore, previous studies report that depression is the main reason for prescribing SSRI antidepressants [57–60], and we previously demonstrated poorer education and health outcomes associated with antidepressants regardless of whether ascertainment was based on the classification described above or whether solely fluoxetine and citalopram were prescribed, the recommended first- and second-line treatments for depression in children and adolescents in the UK [28]. A previous study demonstrated that 62.4% and 62.2% of children prescribed fluoxetine and citalopram did have depression [58]. Medications for ADHD are more specific, but common comorbidities such as oppositional defiant disorder and conduct disorder may still exist, and we could not identify these in our cohort. Our sensitivity analyses, however, did adjust for additional support needs, which includes needs attributed to social, emotional, and behavioural issues. We used existing, administrative databases established for other purposes. However, they undergo regular quality assurance checks, and while the linkage between education and health records used probabilistic matching, we have previously shown this to be 99% accurate for singletons [36]. The study included only local-authority schools, but in Scotland, only 5% of children attend private schools. According to the 2011 Scottish Census, 11% of Scottish residents aged 5–19 years were born outside of Scotland, consistent with the 12.3% of Scottish children we could not link to Scottish maternity records. Prevalence of any (4.3%) and 2 or more (0.6%) of those conditions was similar in pupils who did not link versus those who did (4.7% and 0.6%, respectively). Finally, while we identified whether children did or did not have intellectual disabilities, we had no further information on their level of cognitive abilities.

The introduction of treatment plans is welcome as an attempt to provide children with more personalised healthcare. However, the situation is still not perfect, and more progress is required. There remain aspects of structural organisation and process within healthcare as well as social care and education that are not conducive to holistic management. For example, clinical training, clinical guidelines, and clinical trials are structured around individual conditions, and as such, children are likely to have their conditions managed by different physicians. For example, epilepsy may be treated at a neurology clinic, whereas a community paediatrician or child psychiatrist may treat ADHD. Many charities also still focus on individual conditions such as epilepsy or ASD. Given that these children also experience comorbid physical conditions, a more joined-up approach to care is required. Additionally, further studies could usefully attempt to disentangle whether all children with neurodevelopmental multimorbidity benefit from more comprehensive treatment plans and a more joined-up approach to care, as well as the extent to which any benefits relate to the extent of cognitive abilities of the children in the different multimorbidity groupings. Such studies could inform what interventions are most relevant to increase school attendance, and for which groups of individuals.

## Conclusion

Our study found that, compared to their peers, children with neurodevelopmental multimorbidity fared worse across all of the educational outcomes measured, experiencing greater absenteeism and exclusion, poorer attainment, and increased unemployment even after adjusting for sociodemographic and maternity factors, comorbid physical conditions, and additional support needs. Based on these findings, our study suggests that focussing healthcare systems and training on individual neurodevelopmental conditions may disadvantage children with neurodevelopmental multimorbidity by failing to recognise and address all of their needs. Early diagnosis and earlier intervention extending beyond the health sector into schools is needed to minimise long-term adverse outcomes. This could be in the form of additional support programmes that aim to reduce the risk of adverse outcomes in at-risk groups. We found that the increased risk of poor exam results was in part explained by higher rates of exclusion from school, and that poorer exam results, in turn, explained increased risk of unemployment. We acknowledge that, in our study, we did not know whether children with neurodevelopmental multimorbidity had lower cognitive abilities than children with single conditions, or whether certain groupings of neurodevelopmental multimorbidity were associated with lower cognitive abilities to a greater extent than other groupings. Furthermore, we do not know whether children’s educational and occupational outcomes would benefit from intervention to the same or to a lesser extent if they have lower cognitive abilities. While acknowledging these uncertainties, we conclude that efforts should focus on interventions that can improve academic attainment and reduce absence and exclusion—or their respective impact—on affected children.

## Supporting information

S1 Table Summary outcomes among least and most deprived children with neurodevelopmental multimorbidity and no morbidity. (DOCX)

S1 Fig Flow diagram illustrating the number of pupils included and excluded from the cohort at each stage of data cleaning. (TIF)

S2 Fig Associations between different combinations of neurodevelopmental conditions and attainment. Adjusted for age, sex, deprivation quintile, ethnic group, maternal age, maternal smoking, parity, mode of delivery, gestation at delivery, sex-gestation–specific birth weight centile, and 5-minute Apgar score. All categories referent to children with no conditions. 139,205 pupils analysed using binary logistic regression to produce ORs. Bars represent 95% confidence intervals. All models significant at *p <* 0.001. ADHD, attention deficit hyperactivity disorder; ASD, autism spectrum disorder; D, depression; ID, intellectual disability; OR, odds ratio.
(TIF)

S3 Fig Associations between different combinations of neurodevelopmental conditions and unemployment. Adjusted for age, sex, deprivation quintile, ethnic group, maternal age, maternal smoking, parity, mode of delivery, gestation at delivery, sex-gestation–specific birth weight centile, and 5-minute Apgar score. All categories referent to children with no conditions. 217,924 pupils analysed using binary logistic regression to produce ORs. Bars represent 95% confidence intervals. All models significant at *p <* 0.001 except ^a^*p <* 0.01; ^b^*p <* 0.05; ^c^*p* = 0.051; ^d^*p* = 0.052; ^e^*p* = 0.149; ^f^*p* = 0.419; ^g^*p* = 0.809. ADHD, attention deficit hyperactivity disorder; ASD, autism spectrum disorder; D, depression; ID, intellectual disability; OR, odds ratio.
(TIF)

S1 STROBE Checklist STROBE, Strengthening the Reporting of Observational Studies in Epidemiology. (DOCX)

## Abbreviations

ADHD: attention deficit hyperactivity disorder
ASD: autism spectrum disorder
CHI: Community Health Index
GEE: generalised estimating equation
IRR: incidence rate ratio
ISD: Information Services Division
OCD: obsessive-compulsive disorder
OR: odds ratio
PIS: Prescribing Information System
QIC: quasi-likelihood under the independence model criterion
ScotXed: Scottish Exchange of Educational Data
SCQF: Scottish Credit Qualifications Framework
SIMD: Scottish Index of Multiple Deprivation
SMR: Scottish Morbidity Record
SQA: Scottish Qualifications Authority
SSRI: selective serotonin reuptake inhibitor
STROBE: Strengthening the Reporting of Observational Studies in Epidemiology
