# Supplementary material for: Neurodevelopmental multimorbidity and educational outcomes of Scottish schoolchildren: A population-based record linkage cohort study
Source: PLoS Med. 2020 Oct 13;17(10):e1003290. doi: 10.1371/journal.pmed.1003290 (PMC7553326; doi:10.1371/journal.pmed.1003290)
Supplement: S1 Table — (DOCX) [file pmed.1003290.s001.docx]

|  | No conditions | | Two or more conditions | |
| --- | --- | --- | --- | --- |
|  | most deprived SIMD category | least deprived SIMD category | most deprived SIMD category | least deprived SIMD category |
| Average annual number of days absent | 11.00 | 5.00 | 10.75 | 6.00 |
| Percentage of children ever excluded | 6.56 | 1.18 | 15.92 | 7.84 |
| Percentage of children unemployed | 16.51 | 4.66 | 18.77 | 18.93 |
| Percentage of children obtaining lowest level of attainment | 54.72 | 15.80 | 81.73 | 58.73 |

SIMD Scottish Index of Multiple Deprivation
