# Supplementary figures and images for: Neurodevelopmental multimorbidity and educational outcomes of Scottish schoolchildren: A population-based record linkage cohort study
Source: PLoS Med. 2020 Oct 13;17(10):e1003290. doi: 10.1371/journal.pmed.1003290 (PMC7553326; doi:10.1371/journal.pmed.1003290)

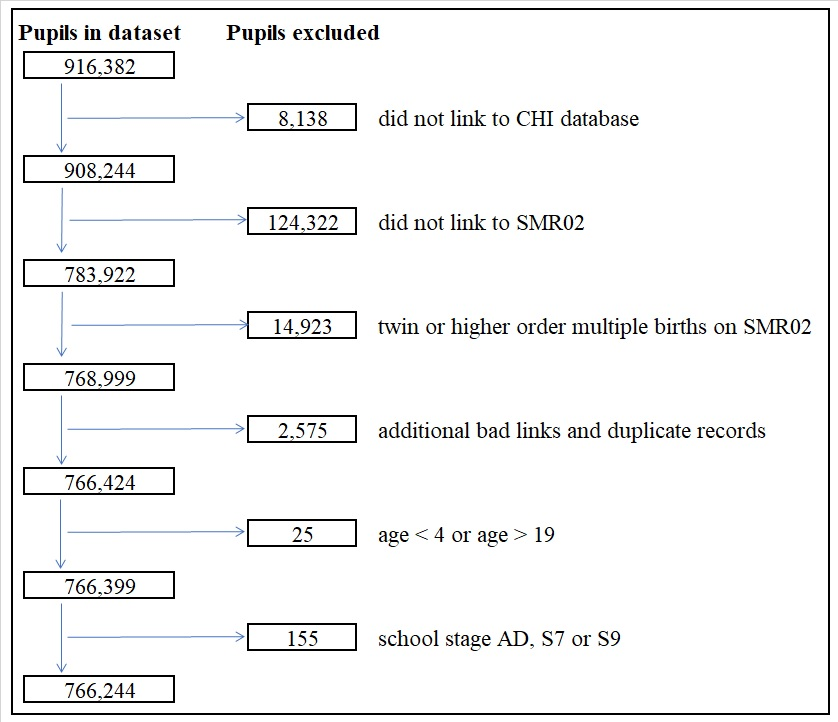

Supplement: S1 Fig — (TIF) [file pmed.1003290.s002.tif]

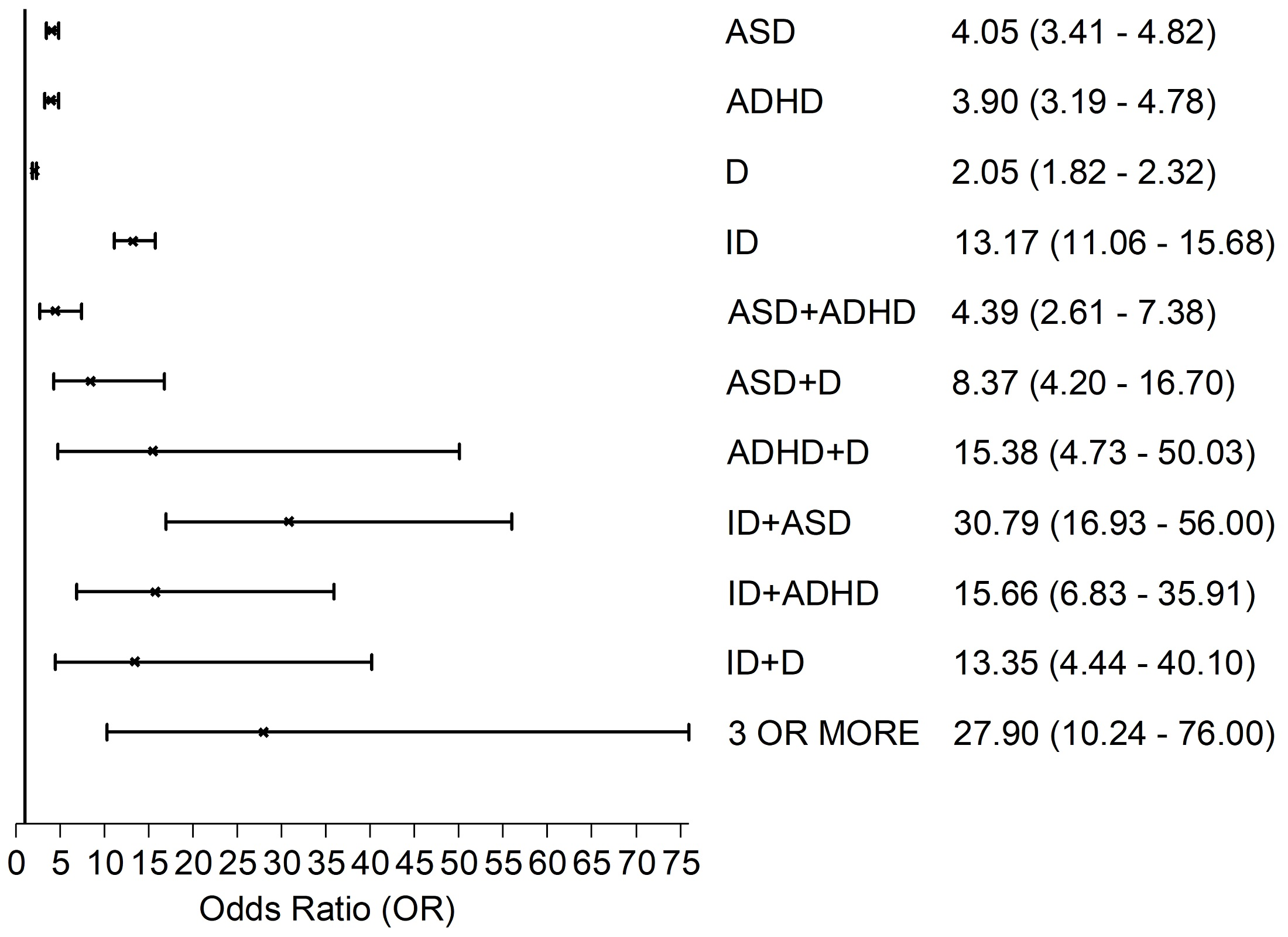

Supplement: S2 Fig — Adjusted for age, sex, deprivation quintile, ethnic group, maternal age, maternal smoking, parity, mode of delivery, gestation at delivery, sex-gestation–specific birth weight centile, and 5-minute Apgar score. All categories referent to children with no conditions. 139,205 pupils analysed using binary logistic regression to produce ORs. Bars represent 95% confidence intervals. All models significant at p < 0.001. ADHD, attention deficit hyperactivity disorder; ASD, autism spectrum disorder; D, depression; ID, intellectual disability; OR, odds ratio. (TIF) [file pmed.1003290.s003.tif]

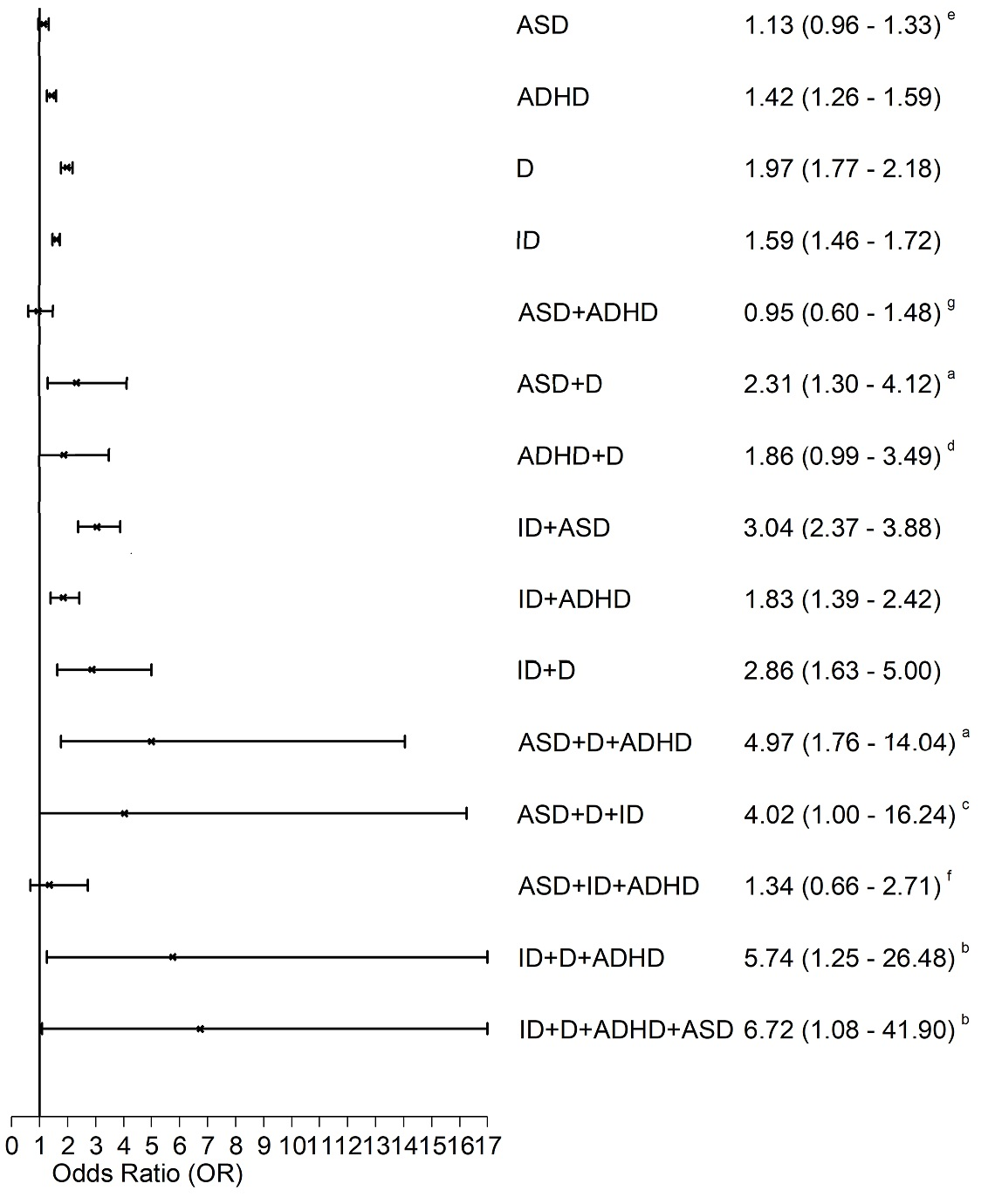

Supplement: S3 Fig — Adjusted for age, sex, deprivation quintile, ethnic group, maternal age, maternal smoking, parity, mode of delivery, gestation at delivery, sex-gestation–specific birth weight centile, and 5-minute Apgar score. All categories referent to children with no conditions. 217,924 pupils analysed using binary logistic regression to produce ORs. Bars represent 95% confidence intervals. All models significant at p < 0.001 except ap < 0.01; bp < 0.05; cp = 0.051; dp = 0.052; ep = 0.149; fp = 0.419; gp = 0.809. ADHD, attention deficit hyperactivity disorder; ASD, autism spectrum disorder; D, depression; ID, intellectual disability; OR, odds ratio. (TIF) [file pmed.1003290.s004.tif]
