# Supplementary material for: Neurodevelopmental multimorbidity and educational outcomes of Scottish schoolchildren: A population-based record linkage cohort study
Source: PLoS Med. 2020 Oct 13;17(10):e1003290. doi: 10.1371/journal.pmed.1003290 (PMC7553326; doi:10.1371/journal.pmed.1003290)
Supplement: S1 STROBE Checklist — (DOCX) [file pmed.1003290.s005.docx]

STROBE Statement—checklist of items that should be included in reports of observational studies

|  | Item No. | Recommendation | Page  No. | Relevant text from manuscript |
| --- | --- | --- | --- | --- |
| **Title and abstract** | 1 | (*a*) Indicate the study’s design with a commonly used term in the title or the abstract |  | Title and first line of Abstract methods and findings |
|  |  | (*b*) Provide in the abstract an informative and balanced summary of what was done and what was found |  | Abstract methods and findings |
| Introduction | | | |  |
| Background/rationale | 2 | Explain the scientific background and rationale for the investigation being reported |  | Second paragraph of introduction |
| Objectives | 3 | State specific objectives, including any prespecified hypotheses |  | Last two sentences of introduction |
| Methods | | | |  |
| Study design | 4 | Present key elements of study design early in the paper |  | Databases section |
| Setting | 5 | Describe the setting, locations, and relevant dates, including periods of recruitment, exposure, follow-up, and data collection |  | Setting, location and relevant dates – ‘Inclusion and exclusion criteria’ section  Recruitment and exposure – ‘Case ascertainment and definition’ section  Data collection – ‘Databases’ and ‘Case ascertainment and definition’ sections |
| Participants | 6 | (*a*) *Cohort study*—Give the eligibility criteria, and the sources and methods of selection of participants. Describe methods of follow-up  *Case-control study*—Give the eligibility criteria, and the sources and methods of case ascertainment and control selection. Give the rationale for the choice of cases and controls  *Cross-sectional study*—Give the eligibility criteria, and the sources and methods of selection of participants |  | Eligibility criteria – ‘Inclusion and exclusion criteria’ section  Selection of patients – ‘Databases’ and ‘Case ascertainment and definition’ sections |
|  |  | (*b*) *Cohort study*—For matched studies, give matching criteria and number of exposed and unexposed  *Case-control study*—For matched studies, give matching criteria and the number of controls per case |  |  |
| Variables | 7 | Clearly define all outcomes, exposures, predictors, potential confounders, and effect modifiers. Give diagnostic criteria, if applicable |  | Exposures and diagnostic criteria - ‘Case ascertainment and definition’ section  Outcomes – ‘Outcomes’ section  Predictors – first paragraph of ‘Statistical analyses’ section  Confounders – ‘Covariates’ section  Effect modifiers and mediators – ‘Statistical analyses’ section |
|  |  |  |  |  |
| Data sources/ measurement | 8* | For each variable of interest, give sources of data and details of methods of assessment (measurement). Describe comparability of assessment methods if there is more than one group |  | Exposures - ‘Case ascertainment and definition’ section  Outcomes – ‘Outcomes’ section  Predictors – first paragraph of ‘Statistical analyses’ section  Confounders – ‘Covariates’ section |
| Bias | 9 | Describe any efforts to address potential sources of bias |  | ‘Statistical analyses’ section |
| Study size | 10 | Explain how the study size was arrived at |  | ‘Inclusion and exclusion criteria’ section |

Continued on next page

| Quantitative variables | 11 | Explain how quantitative variables were handled in the analyses. If applicable, describe which groupings were chosen and why |  | ‘Statistical analyses’ section |
| --- | --- | --- | --- | --- |
| Statistical methods | 12 | (*a*) Describe all statistical methods, including those used to control for confounding |  | ‘Statistical analyses’ section |
|  |  | (*b*) Describe any methods used to examine subgroups and interactions |  | ‘Statistical analyses’ section |
|  |  | (*c*) Explain how missing data were addressed |  | Results section, Discussion section and Supplementary Figure 1 |
|  |  | (*d*) *Cohort study*—If applicable, explain how loss to follow-up was addressed  *Case-control study*—If applicable, explain how matching of cases and controls was addressed  *Cross-sectional study*—If applicable, describe analytical methods taking account of sampling strategy |  | N/A |
|  |  | (*e*) Describe any sensitivity analyses |  | ‘Statistical analyses’ section |
| Results | | | | |
| Participants | 13* | (a) Report numbers of individuals at each stage of study—eg numbers potentially eligible, examined for eligibility, confirmed eligible, included in the study, completing follow-up, and analysed |  | Supplementary Figure 1 |
|  |  | (b) Give reasons for non-participation at each stage |  | N/A |
|  |  | (c) Consider use of a flow diagram |  | Supplementary Figure 1 |
| Descriptive data | 14* | (a) Give characteristics of study participants (eg demographic, clinical, social) and information on exposures and potential confounders |  | Table 1 |
|  |  | (b) Indicate number of participants with missing data for each variable of interest |  | Table 1 |
|  |  | (c) *Cohort study*—Summarise follow-up time (eg, average and total amount) |  | Abstract methods and findings section |
| Outcome data | 15* | *Cohort study*—Report numbers of outcome events or summary measures over time |  | Results section and supplementary table 1 |
|  |  | *Case-control study—*Report numbers in each exposure category, or summary measures of exposure |  |  |
|  |  | *Cross-sectional study—*Report numbers of outcome events or summary measures |  |  |
| Main results | 16 | (*a*) Give unadjusted estimates and, if applicable, confounder-adjusted estimates and their precision (eg, 95% confidence interval). Make clear which confounders were adjusted for and why they were included |  | Covariates section within Methods  Results section  Tables 3 and 4, Figures 1 and 2, and Supplementary Figures 1 and 2 |
|  |  | (*b*) Report category boundaries when continuous variables were categorized |  | Table 1 |
|  |  | (*c*) If relevant, consider translating estimates of relative risk into absolute risk for a meaningful time period |  |  |

Continued on next page

| Other analyses | 17 | Report other analyses done—eg analyses of subgroups and interactions, and sensitivity analyses |  | Results section, Table 3 and Table 4 |
| --- | --- | --- | --- | --- |
| Discussion | | | | |
| Key results | 18 | Summarise key results with reference to study objectives |  | First paragraph of Discussion section |
| Limitations | 19 | Discuss limitations of the study, taking into account sources of potential bias or imprecision. Discuss both direction and magnitude of any potential bias |  | Discussion section |
| Interpretation | 20 | Give a cautious overall interpretation of results considering objectives, limitations, multiplicity of analyses, results from similar studies, and other relevant evidence |  | Discussion section |
| Generalisability | 21 | Discuss the generalisability (external validity) of the study results |  | Discussion section |
| Other information | |  | | |
| Funding | 22 | Give the source of funding and the role of the funders for the present study and, if applicable, for the original study on which the present article is based |  | Funding and Acknowledgements sections |

*Give information separately for cases and controls in case-control studies and, if applicable, for exposed and unexposed groups in cohort and cross-sectional studies.

**Note:** An Explanation and Elaboration article discusses each checklist item and gives methodological background and published examples of transparent reporting. The STROBE checklist is best used in conjunction with this article (freely available on the Web sites of PLoS Medicine at http://www.plosmedicine.org/, Annals of Internal Medicine at http://www.annals.org/, and Epidemiology at http://www.epidem.com/). Information on the STROBE Initiative is available at www.strobe-statement.org.
